# *Hericium erinaceus* Mycelium Ameliorates In Vivo Progression of Osteoarthritis

**DOI:** 10.3390/nu14132605

**Published:** 2022-06-23

**Authors:** Shang-Yu Yang, Chi-Jung Fang, Yu-Wen Chen, Wan-Ping Chen, Li-Ya Lee, Chin-Chu Chen, Yen-You Lin, Shan-Chi Liu, Chun-Hao Tsai, Wei-Chien Huang, Yang-Chang Wu, Chih-Hsin Tang

**Affiliations:** 1 Department of Healthcare Administration, College of Medical and Health Science, Asia University, Taichung 41354, Taiwan; henry879019@yahoo.com.tw; 2 Department of Orthopaedic Surgery, An Nan Hospital, China Medical University, Tainan 40447, Taiwan; ed107087@gmail.com; 3 Biotech Research Institute, Grape King Bio Ltd., Taoyuan 325002, Taiwan; annie.chen@grapeking.com.tw (Y.-W.C.); wp.chen@grapeking.com.tw (W.-P.C.); ly.lee@grapeking.com.tw (L.-Y.L.); 4 Institute of Food Science and Technology, National Taiwan University, Taipei 106617, Taiwan; gkbioeng@grapeking.com.tw; 5 Department of Food Science, Nutrition and Nutraceutical Biotechnology, Shih Chien University, Taipei 104, Taiwan; 6 Department of Bioscience Technology, Chung Yuan Christian University, Taoyuan 320314, Taiwan; 7 Department of Pharmacology, School of Medicine, China Medical University, Taichung 404333, Taiwan; chas6119@gmail.com; 8 Department of Medical Education and Research, China Medical University Beigang Hospital, Yunlin 651012, Taiwan; sdsaw.tw@yahoo.com.tw; 9 Department of Orthopedic Surgery, China Medical University Hospital, Taichung 404333, Taiwan; ritsai8615@gmail.com; 10 Department of Sports Medicine, College of Health Care, China Medical University, Taichung 404333, Taiwan; 11 Graduate Institute of Biomedical Sciences, China Medical University, Taichung 404333, Taiwan; whuang@mail.cmu.edu.tw; 12 Drug Development Center, China Medical University, Taichung 404333, Taiwan; 13 Department of Chinese Medicine, China Medical University Hospital, Taichung 404333, Taiwan; yachwu@mail.cmu.edu.tw; 14 Chinese Medicine Research and Development Center, China Medical University Hospital, China Medical University, Taichung 404333, Taiwan; 15 Chinese Medicine Research Center, China Medical University, Taichung 404333, Taiwan; 16 Department of Medical Laboratory Science and Biotechnology, Asia University, Taichung 41354, Taiwan

**Keywords:** osteoarthritis, *Hericium erinaceus*, mycelium, anterior cruciate ligament transection, interleukin 1 beta, tumor necrosis factor-alpha

## Abstract

Osteoarthritis (OA) is an age-related disorder that affects the joints and causes functional disability. *Hericium erinaceus* is a large edible mushroom with several known medicinal functions. However, the therapeutic effects of *H. erinaceus* in OA are unknown. In this study, data from Sprague-Dawley rats with knee OA induced by anterior cruciate ligament transection (ACLT) indicated that *H. erinaceus* mycelium improves ACLT-induced weight-bearing asymmetry and minimizes pain. ACLT-induced increases in articular cartilage degradation and bone erosion were significantly reduced by treatment with *H. erinaceus* mycelium. In addition, *H. erinaceus* mycelium reduced the synthesis of proinflammatory cytokines interleukin-1β and tumor necrosis factor-α in OA cartilage and synovium. *H. erinaceus* mycelium shows promise as a functional food in the treatment of OA.

## 1. Introduction

Osteoarthritis (OA) is an age-related disorder that affects the joints and causes functional disability [1]. During OA development, low-grade inflammatory reactions progressively degrade the joints [2]. Typical OA symptoms include joint swelling and deformities that are associated with constant pain and consequent interference with normal daily life activities [2]. Around 80% of OA patients face movement disorders, 20% cannot perform basic activities and 10% require daily care [3]. The ever-growing numbers of elderly people worldwide are confounding the already large healthcare and economic burdens imposed by patients with OA [2,4].

Two major proinflammatory cytokines, interleukin 1 beta (IL-1β) and tumor necrosis factor-alpha (TNF-α), facilitate the development of OA by increasing catabolic enzyme formation that degrades the cartilage extracellular matrix [5,6]. Levels of IL-1β and TNF-α expression are higher in human OA serum and synovial fluid than in samples from healthy individuals [7,8], and they are targeted by therapies such as the anti-IL-1β antibody canakinumab and the TNF-α-blocking agent adalimumab [7]. Inhibiting proinflammatory cytokine expression successfully inhibits OA progression [5,9].

Nonsteroidal anti-inflammatory drugs (NSAIDs) and corticosteroids are commonly applied to inhibit ongoing inflammation and reduce the pain associated with OA [10,11]. However, the undesirable side effects of these synthetic agents make the discovery of anti-OA ingredients from natural products an attractive proposition. *Hericium erinaceus* is a large edible mushroom that is popularly consumed in Asian countries and is accepted as a dietary supplement or functional food [12,13]. *H. erinaceus* is rich in bioactive compounds including glycoproteins, polysaccharides and ketones [14]. In addition, the mushroom fruiting bodies, mycelium and bioactive pure compounds of *H. erinaceus* exhibit several medicinal functions including antitumor, anti-inflammatory, nephroprotective, neuroprotective effects, antimicrobial, antioxidant, immunomodulatory and antihyperglycemic properties [12,15,16,17,18,19,20]. A standardized extract containing *H. erinaceus* (Bull.) Persoon, *Kalopanax pictus* Castor-Aralia and *Astragalus membranaceus* Schischkin has shown in vitro and in vivo chondroprotective effects in OA models [21]. However, the therapeutic effects of *H. erinaceus* in human OA remain unknown. Here, we found that *H. erinaceus* mycelium prevents disease progression in an anterior cruciate ligament transection (ACLT) model of OA, suggesting that *H. erinaceus* mycelium has therapeutic utility for OA.

## 2. Materials and Methods

### 2.1. Preparation of Hericium erinaceus Mycelium

The Bioresource Collection and Research Center (BCRC, Food Industry Research and Development Institute, Hsinchu, Taiwan) supplied *H. erinaceus* mycelium (BCRC strain no. 35669) [22]. The strain was first grown in a potato dextrose agar plate at 25 °C for 15 days. The *H. erinaceus* mycelium cultures were transferred to 1.3 L of liquid medium in 2 L flasks and cultured for five days at 25 °C in a shaking incubator at 120 rpm. The cultures were scaled-up to a 500-L bioreactor for a further five days, then to a 20-ton fermenter for 12 days, under the same conditions described above. The culture liquid medium used for scaling-up was adjusted to pH 4.5 and contained 4.5% glucose, 0.5% soybean powder, 0.25% yeast extract, 0.25% peptone and 0.05% MgSO_4_. Finally, *H. erinaceus* mycelium from the 20-ton fermentation process were harvested, lyophilized and ground into powder. The dosage of *H. erinaceus* mycelium applied in the OA animal model was equivalent to a 60 kg adult consuming 1 g of *H. erinaceus* powder daily.

### 2.2. Anterior Cruciate Ligament Transection (ACLT) Animal Model

Male Sprague-Dawley (SD) rats (eight weeks of age; 300–350 g) were purchased from the National Laboratory Animal Center (Taipei, Taiwan) and randomly divided into three groups: sham surgery (controls), ACLT only and ACLT with *H. erinaceus* (100 mg/kg). ACLT surgery was performed according to the procedure mentioned in our previous reports [6,23]. Briefly, the rats were anesthetized and underwent arthrotomy to expose the right knee joint, and the ACL was severed. Controls underwent arthrotomy only. Two days after surgery, the rats started to receive *H. erinaceus* mycelium. The static weight-bearing incapacitance test (Bioseb, Paris, France) evaluated spontaneous pain after ACLT, as previously described [24]. The left and right hind limbs were placed on separate sensor plates to measure between-limb differences in dynamic weight bearing (expressed as g) over a 10-s period. The mean score of three consecutive measurements was recorded for each animal on every test day.

### 2.3. Micro-Computed Tomography (μ-CT) Measurements

Micro-CT analysis was performed at six weeks after ACLT surgery. The rats were sacrificed and the right knee joints were collected and fixed in 4% formaldehyde and then 70% ethanol at room temperature, as previously described [17,25]. The knee joints were scanned by a SkyScan 2211 micro-CT system (Bruker, Kontich, Belgium), using a voxel resolution of 10.5 µm over 180° of rotation, a voltage of 70 kVp, a current of 290 µA and a 0.5-mm aluminum filter to prevent beam-hardening artifacts. Image reconstruction of coronal and transverse images used InstaRecon^®^ software (Bruker micro-CT, Kontich, Belgium). Reconstructed cross-sections were reorientated and 59 slices (0.5 mm) were selected, then manual regions of interest (ROI) were drawn. Bone mineral density (BMD), bone mineral content (BMC), bone volume/total volume (BV/TV), bone surface/total volume (BS/TV), trabecular thickness (Tb.Th), trabecular number (Tb.N) and trabecular separation (Tb.Sp) were analyzed by Bruker micro-CT software (CTAn, version 1.7.1, Bruker, Kontich, Belgium), as previously detailed [26,27].

### 2.4. Immunohistochemistry (IHC)

The right knee joints were decalcified in 10% EDTA of phosphate-buffered saline for two weeks after μ-CT scanning. The knee samples were then dehydrated with ethanol (from 70% to 100%) and embedded in paraffin blocks to prepare slices of 5-µm thicknesses. Hematoxylin & Eosin (H&E) and Safranin-O/Fast Green staining enabled us to analyze histopathological changes under an optical microscope, as previously described [27,28]. For analysis of IL-1β and TNF-α expression, the tissue sections were stained with primary antibodies against IL-1β or TNF-α (GeneTex; Hsinchu, Taiwan) at 4 °C overnight, followed by incubation with secondary antibody (1:200) at room temperature for 1 h. The sections were stained with diaminobenzidine and observed under a light microscope, as previously described [29,30]. The sum of the intensity and percentage scores was used as the final staining score [25].

### 2.5. Statistical Analysis

All values are given as the mean ± standard deviation (SD). The statistical calculations were analyzed by using PRISM 5.0 software (GraphPad, San Diego, CA, USA). The paired sample *t*-test was selected to compare results from two groups. One-way ANOVA followed by Bonferroni post-hoc testing for multiple comparisons was used to analyze more than two groups. Student’s *t*-test assessed between-group differences. A *p*-value of <0.05 was considered statistically significant.

## 3. Results

### 3.1. H. erinaceus Mycelium Reduces ACLT-Induced Weight-Bearing Asymmetry and Pain

**Figure 1:**
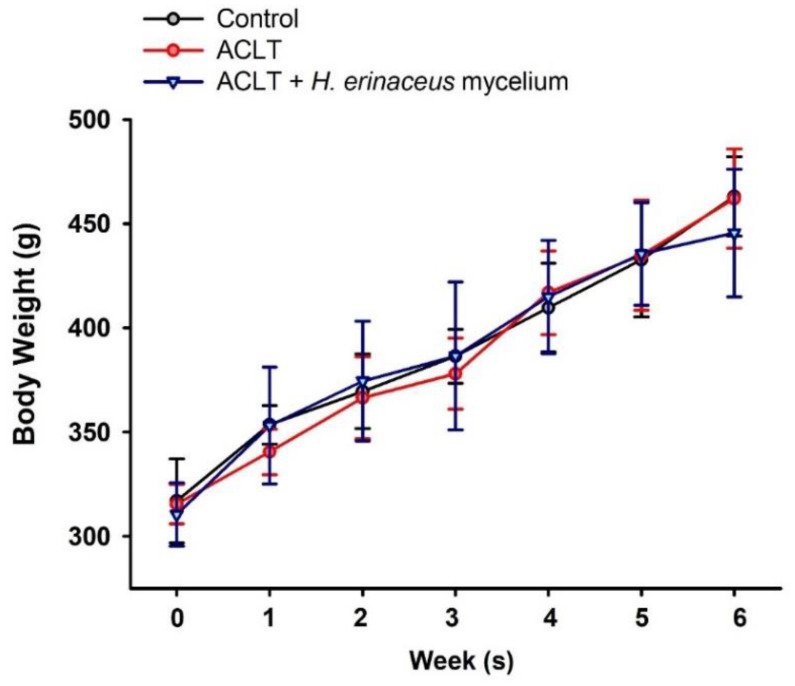
Body weight gain during the experimental period. Body weight was measured during the experimental period. (*n* = 6 for each group).

**Figure 2:**
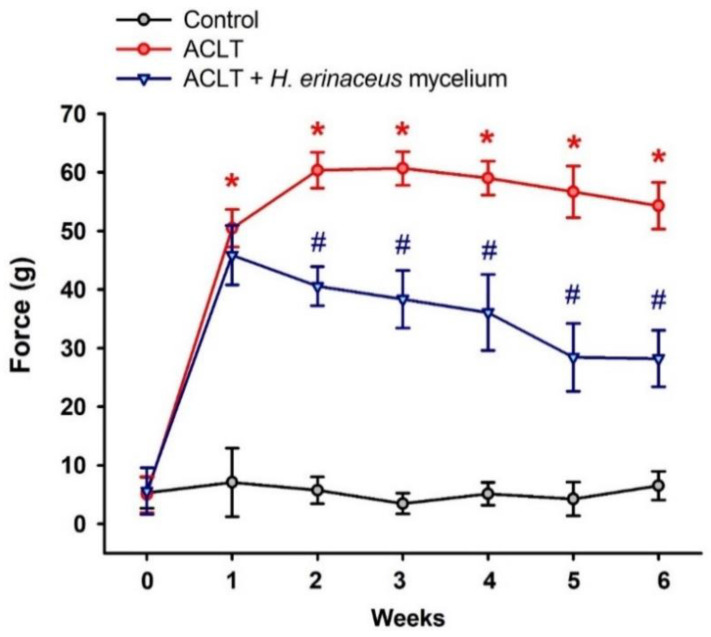
*H. erinaceus* mycelium improves ACLT-induced weight-bearing asymmetry. Deficits in weight-bearing forces were examined every week by weight-bearing behavioral testing (*n* = 6 for each group). * *p* < 0.05 compared to the control group; # *p* < 0.05 compared to the ACLT-only group.

We examined the effects of *H. erinaceus* mycelium in a rat model of ACLT [6,23]. As shown in Figure 1, no changes in body weight were observed in the ACLT-only and ACLT + *H. erinaceus* groups. At six weeks, rats fed with *H. erinaceus* mycelium exhibited significant improvements in ACLT-induced weight-bearing asymmetry and pain compared to the ACLT-only group (Figure 2).

### 3.2. H. erinaceus Mycelium Improves Bone and Cartilage Architecture in ACLT Rats

**Figure 3:**
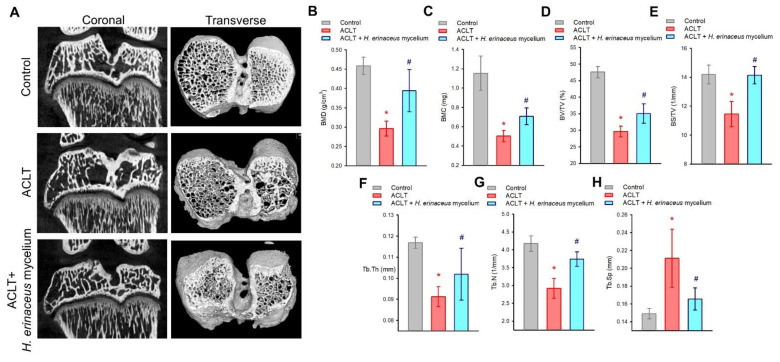
Micro-CT analysis of the effects of *H. erinaceus* mycelium on the ACLT bone architecture. (**A**) Representative micro-CT images from knee subchondral bone. Quantitative analyses of (**B**) BMD, (**C**) BMC, (**D**) BV/TV, (**E**) BS/TV, (**F**) Tb.Th, (**G**) Tb.N and (**H**) Tb.Sp (*n* = for each group). * *p* < 0.05 compared to the control group; # *p* < 0.05 compared to the ACLT-only group.

Next, we used μ-CT to analyze in detail the changes in bone and cartilage architecture after *H. erinaceus* mycelium application. Marked improvements were seen in bone mineral density (BMD), bone mineral content (BMC), bone volume/tissue volume (BV/TV), bone surface/TV (BS/TV), trabecular thickness (Tb.Th), trabecular number (Tb.N) and trabecular separation (Tb.Sp), compared to the ACLT-only group (Figure 3).

**Figure 4:**
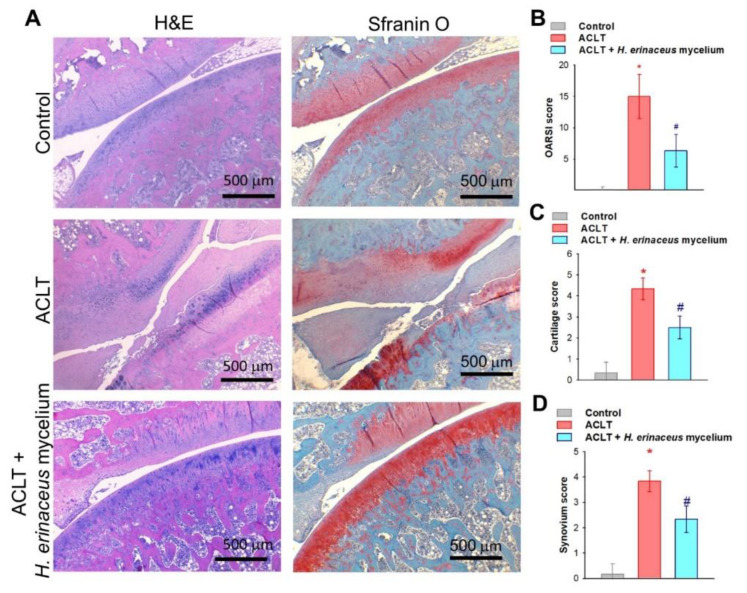
*H. erinaceus* mycelium ameliorates ACLT-induced cartilage degradation and synovial inflammation. (**A**) Histological sections from knees stained with H&E and Safranin-O. (**B**) OARSI scores, (**C**) cartilage scores and (**D**) synovium scores (*n* = 6 for each group). * *p* < 0.05 compared to the control group; # *p* < 0.05 compared to the ACLT-only group.

H&E and Safranin-O staining revealed that *H. erinaceus* mycelium dramatically prevented ACLT-induced increases in Osteoarthritis Research Society International (OARSI) scores, cartilage and synovium scores and cartilage damage (Figure 4).

### 3.3. H. erinaceus Mycelium Suppresses Proinflammatory Cytokine Upregulation

**Figure 5:**
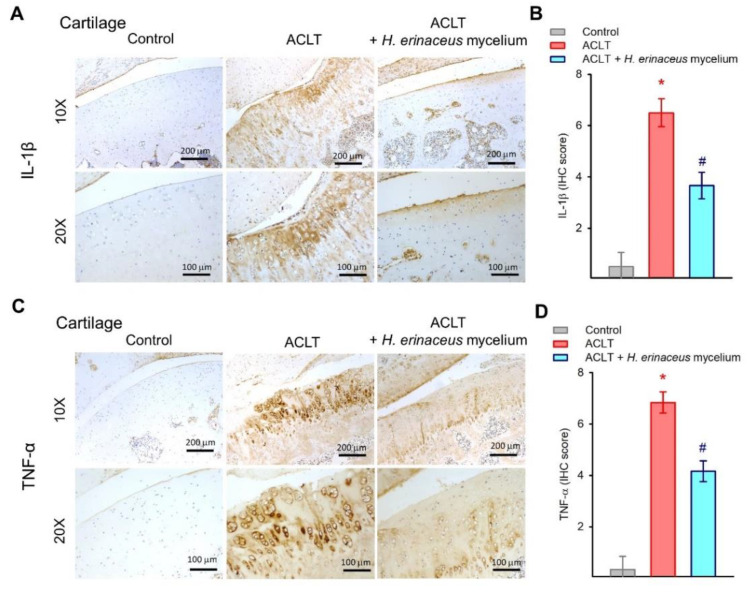
*H. erinaceus* mycelium suppresses IL-1β and TNF-α expression in the cartilage. (**A**,**C**) Representative images of IL-1β and TNF-α staining. (**B**,**D**) Quantification of IHC scores (*n* = 6 for each group). * *p* < 0.05 compared to the control group; # *p* < 0.05 compared to the ACLT-only group.

**Figure 6:**
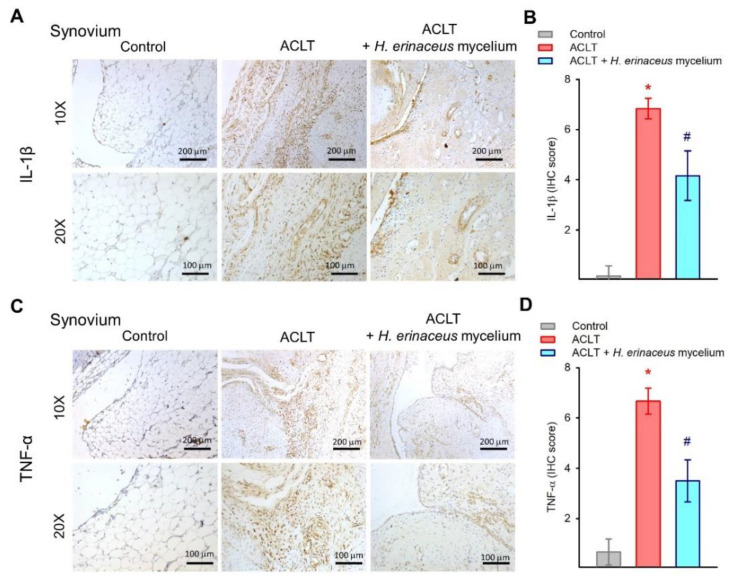
*H. erinaceus* mycelium inhibits IL-1β and TNF-α expression in synovial tissue. (**A**,**C**) Representative images of IL-1β and TNF-α staining. (**B**,**D**) Quantification of IHC scores (*n* = 6 for each group). * *p* < 0.05 compared to the control group; # *p* < 0.05 compared to the ACLT-only group.

IL-1β and TNF-α are critical proinflammatory cytokines during OA progression [5,6]. IHC staining revealed that while IL-1β and TNF-α synthesis was significantly elevated in the cartilage and synovium of the ACLT-only group, the expression of both cytokines in both tissues was lowered by *H. erinaceus* mycelium (Figure 5 and Figure 6).

## 4. Discussion

OA causes great physical disability [31]. Much remains unknown about the pathogenesis of OA, although synovial inflammation is a well-recognized factor [32], so treating synovial inflammation is favored as an effective means of inhibiting the progression of OA [33,34]. Elevated levels of proinflammatory cytokine expression are found in OA joints [35]. The ACLT animal model for surgical initiation of OA erodes the knee joint cartilage, leading to OA-like disease [6,23]. Here, we found that *H. erinaceus* mycelium antagonized ACLT-induced promotion of weight-bearing asymmetry, bone loss, synovial inflammation and degradation of articular cartilage. In addition, *H. erinaceus* mycelium effectively reduced IL-1β and TNF-α levels in cartilage and synovial tissue, suggesting promising therapeutic effects for OA.

Numerous proinflammatory mediators are produced during the progression of OA, including IL-1β and TNF-α, leading to the activation of catabolic factors, resulting in cartilage degradation and bone erosion [36,37]. The levels of IL-1β and TNF-α expression in serum and synovial tissue are associated with the pathological process of OA [38,39]. Our ACLT-induced OA model demonstrated that ACLT surgery mimics clinical features, increasing IL-1β and TNF-α synthesis in cartilage and synovial tissue. *H. erinaceus* mycelium administration clearly downregulated the IL-1β and TNF-α expression in both cartilage and synovial tissues. The anti-OA effects of *H. erinaceus* mycelium are due to its ability to inhibit IL-1β and TNF-α production.

Nonsurgical treatment OARSI guidelines issued in 2019 suggest that exercises such as balance training and muscle strengthening are important components in the control of OA [4]. In regard to pharmacological therapy, the OARSI guidelines strongly recommend (Level 1A evidence) topical NSAIDs for patients with knee OA, while intra-articular hyaluronic acid or intra-articular corticosteroids are recommended (Level 1B/Level 2) treatments for knee OA dependent on comorbidities. Oral NSAIDs are conditionally not recommended (Levels 4A and 4B) and oral or transdermal opioids are strongly not recommended (Level 5) [4]. Although NSAIDs are commonly used for OA patients, these agents have unwanted side effects including substantial damage to the gastrointestinal and cardiovascular systems [40]. Our results indicate that *H. erinaceus* mycelium prevents cartilage damage by inhibiting ACLT-facilitated promotion of OARSI, cartilage and synovium scores. Our study data show that OA-induced damage to bone microarchitectural parameters BMD, BMC, BV/TV, BS/TV, Tb.Th and Tb.N was rescued by treatment with *H. erinaceus* mycelium. Thus, *H. erinaceus* mycelium protects against cartilage degradation and bone erosion.

Natural products have been used to remedy human disorders for millennia, and *H. erinaceus* is a well-known component of traditional Chinese medicine [41,42]. The constituents of *H. erinaceus* have been examined, and their functions have been documented for different body systems, particularly the nervous system [43,44]. *H. erinaceus* contains many bioactive components such as polysaccharides, secondary metabolites and nutritional components [45]. Several important polyphenol oxidase inhibitors (adenosine, ergosterol, ergothioneine and glutathione) have been found in *H. erinaceus* mycelium [46]. Other research has also identified that the diterpenoids Erinacine A and Erinacine S show high levels of bioactivity in *H. erinaceus* extract, after analysis by HPLC and LC-MS methods [47,48,49,50]. However, whether any of these bioactive compounds possess anti-arthritic functions remains to be investigated. We have identified a novel function for *H. erinaceus* mycelium as an effective inhibitor of ACLT-induced facilitation of weight-bearing asymmetry and pain, cartilage degradation, bone erosion and proinflammatory cytokine production. Thus, *H. erinaceus* mycelium may serve as a functional food that is beneficial in OA therapy.

## Data Availability

The raw data for this study are available from the corresponding authors on reasonable request.
